# COMPARISON OF CUT POINTS FOR PREDICTING PHYSICAL ACTIVITY INTENSITY IN OLDER ADULTS

**DOI:** 10.1093/geroni/igad104.2306

**Published:** 2023-12-21

**Authors:** Ameera Chakravarthy, Barbara Resnick, Elizabeth Galik, Kelly Doran, Cynthia Renn, Eric Lehr, Kasey Knopp

**Affiliations:** University of Maryland School of Nursing, Baltimore, Maryland, United States; University of Maryland, Baltimore, Maryland, United States; University of Maryland School of Nursing, Baltimore, Maryland, United States; UMSON, Baltimore, Maryland, United States; University of Maryland School of Nursing, Baltimore, Maryland, United States; Swedish Heart and Vascular Institute Swedish Medical Center, Seattle, Washington, United States; University of Maryland School of Medicine, Baltimore, Baltimore, Maryland, United States

## Abstract

The differences between cut-points, and scarcity of comparative impact studies have prevented researchers from reaching consensus regarding the application of intensity-related accelerometer cut-points for older adults. This study will focus on the measure of physical activity using the MotionWatch 8 in older adults and compare the differences using traditional Freedson cut points versus those developed by Landry for older adults and evaluate the impact of these differences on age, gender, race, cognition, comorbidities as well as function based on the Barthel Index. A total of 177 participants between the ages of 59 to 101 (M 83) from an acute care study were used for the analysis. Physical activity intensity was estimated using 24-hour count data and compared using two independently developed cut-points. Impact on outcomes were evaluated with hierarchical regression and Multivariate analysis of variance. After controlling for age, gender, race, comorbidities, and cognition, Landry sedentary cut-points were all significantly associated with function. Overall, 14% of the variance in function was explained by all the variables in the model. The findings provide support for use of the Landry cut points to quantify physical activity intensity in hospitalized older adults living with dementia to identify low levels of activity.

